# From weak to strong coupling: quasi-BIC metasurfaces for mid-infrared light–matter interactions

**DOI:** 10.1515/nanoph-2024-0043

**Published:** 2024-04-15

**Authors:** Shovasis Kumar Biswas, Wihan Adi, Aidana Beisenova, Samir Rosas, Eduardo Romero Arvelo, Filiz Yesilkoy

**Affiliations:** Department of Electrical and Computer Engineering, 5228 University of Wisconsin-Madison, Madison, WI 53706, USA; Department of Biomedical Engineering, 5228 University of Wisconsin-Madison, Madison, WI 53706, USA

**Keywords:** all-dielectric metasurface, bound states in the continuum (BIC), vibrational strong coupling, cavity-coupled light–matter interactions

## Abstract

Thanks to their giant, yet tunable, *Q*-factor resonances, all-dielectric metasurfaces supporting the quasi-bound states in the continuum (q-BIC) resonances are well-suited to provide a promising platform for quantum-coherent light–matter interactions. Yet, the strong coupling regime, characterized by the hybrid light–matter states – polaritons, has not yet been fully explored in the mid-infrared regime. This paper investigates the parameter space of vibrational strong coupling (VSC) between material and metasurface cavities supporting q-BIC resonances in the mid-infrared spectral range. We outline the effects of transition dipole strength, damping rate, and the number of molecules coupled to a single cavity, as well as the cavity damping rates, to understand their respective impacts on VSC. By tuning the *Q*-factor of the metasurface and material parameters, a new transition light–matter coupling zone is introduced, bridging the gap between weak and strong coupling, where polaritons form but their linewidths prohibit their spectral identification. The study further identifies the effects of cavity linewidth on polariton peak separability in strongly coupled systems, highlighting that the cavities with smaller nonradiative losses and narrower linewidths facilitate better polariton separability. Moreover, we found that matching cavity and material loss, satisfying the critical strong coupling condition, enhances the coupling strength between cavity and material. Overall, these findings can guide the design of photonic cavities suited for VSC experiments, contributing to the burgeoning fields of polaritonic chemistry, light-mediated modulation of chemical reactivity, and highly sensitive molecular spectroscopy.

## 1 Introduction

Photonic metasurfaces present tremendous opportunities in optics and photonics via efficient spatial and temporal control of light at ultrathin engineered interfaces. A significant development in this domain is the emergence of all-dielectric metasurfaces made from structured high-index dielectric or semiconductor materials with low energy dissipation [1]. Notably, dielectric metasurfaces can confine light intensely into the near-field hotspots, presenting remarkable opportunities for the subwavelength control of light–matter interactions [2], [3], [4]. Specifically, resonances that are based on the bound states in the continuum (BIC) light trapping mechanism can generate accessible and powerful photonic nanocavities on the metasurfaces [5], [6]. Among various photonic structures that can support BICs, metasurfaces built using meta-atoms with broken in-plane inversion symmetry can substantially suppress the radiative losses, leading to sharp resonances associated with quasi-BIC modes (q-BICs) [7]. Across the electromagnetic spectrum, enhanced light–matter interactions supported by q-BIC modes have enabled bourgeoning applications in biochemical sensing [8], [9], [10], surface-enhanced infrared absorption spectroscopy (SEIRAS) [11], THz detection [12], [13], nonlinear light generation [14], [15], enhancing fluorescence [16], photoluminescence [17], and chiral optical response [18]. To date, metasurface applications have usually leveraged light–matter interactions in the weak coupling regime. Exciting technological prospects of quantum-coherent strong light–matter interactions using metasurface photonic cavities are yet to be fully explored.

In the strong coupling regime, the coupling strength *g* between a cavity mode and a molecular transition exceeds the system’s total energy dissipation rates $\left(g>\sqrt{\left(\gamma_{c}^{2}+\gamma_{m}^{2}\right)/2}\right)$ . This leads to a recurrent coherent energy exchange between the cavity and molecular transition modes. The strongly coupled light–matter hybrid system is characterized by new energy states separated by the Rabi energy constant (ℏΩ). A frequency domain analysis can reveal the spectrum of hybrid light–matter states called polaritons, which are quasiparticles simultaneously associated with light and molecules’ transitions.

A photonic cavity’s resonance mode can be designed to strongly couple to electronic or vibrational transitions in a molecule, generating exciton–polaritons or vibro-polaritons, respectively [19]. Since its first demonstration by Yakovley et al. in 1975 [20], strong exciton–photon coupling has been extensively studied using polymers [21], dye molecules [22], quantum dots [23], and transition metal dichalcogenides [24], etc., enabling the observation of some profound effects, including Bose–Einstein condensation [21], and superfluidity [25]. Vibrational strong coupling (VSC), on the other hand, was only recently experimentally demonstrated in 2015 [26], [27], [28]. Since then, there has been a tremendous interest in VSC due to its exciting potential to modify the chemical reactivity of matter. However, achieving VSC is more challenging than strong coupling to electronic states, which are associated with high energy transitions (1–3 eV) and involve electrons delocalizing the entire molecule. Vibrational transitions, however, have lower energy (∼30–500 meV) and are localized to specific bonds in a molecule. This highlights the critical need to develop superior light-harnessing cavities to enable investigations of VSC at the molecular level.

The key prerequisites for cavity-coupled VSC include low photon loss (high-Q or small linewidth) in both the cavity and material, strong resonance light confinement (small mode volumes), and ease of cavity resonance frequency tunability to specific vibrational bands. Additionally, open cavities that enable easy sample access to the photonic modes are crucial for broad photochemical investigations. The Fabry–Perot (FP) cavities with metallic mirrors are the most extensively used systems to explore VSC due to their easy fabrication, simple resonance tuning by gap distance control, robust resonances, and straightforward spectral dispersion analysis by angle-resolved measurements [26], [28], [29]. However, FP cavities are limited in supporting high-Q resonances due to metallic losses of mirrors, and their large mode volumes can only support collective strong coupling to a large number of molecules. Other viable routes to VSC include coupling molecular transitions to propagating surface plasmon polaritons (SPP) [30], [31], and localized surface plasmons supported by nanoantenna with lower mode volumes than the FP cavities [32], [33], [34], [35], [36]. However, plasmonic resonances suffer from ohmic losses inherent to metallic resonators, and the resultant high damping rates hinder their strong coupling to vibrational transitions. Distributed Bragg Reflectors (DBRs)-based mirrors, on the other hand, have high *Q*-factor, but their large mode volume and closed configuration render them typically unsuitable for VSC.

Recently, the strong light-localization capabilities of high-Q dielectric metasurface supporting q-BIC resonances have been employed for VSC [37], [38], [39]. However, the exploration of these VSC systems has been limited to varying film thickness of a single material, polymethyl methacrylate (PMMA), on metasurfaces with comparable material and cavity resonance spectral widths. There is a lack of generalized parameter space investigation that is essential for understanding the interactions between resonant cavity modes and resonant vibrational transitions of molecules at mid-infrared frequencies. To advance VSC-based chemistry using the powerful high-Q metasurface cavities, a comprehensive understanding of the individual roles of cavity and material resonance states is necessary to guide photonic cavity design tailored for different materials.

**Figure 1:**
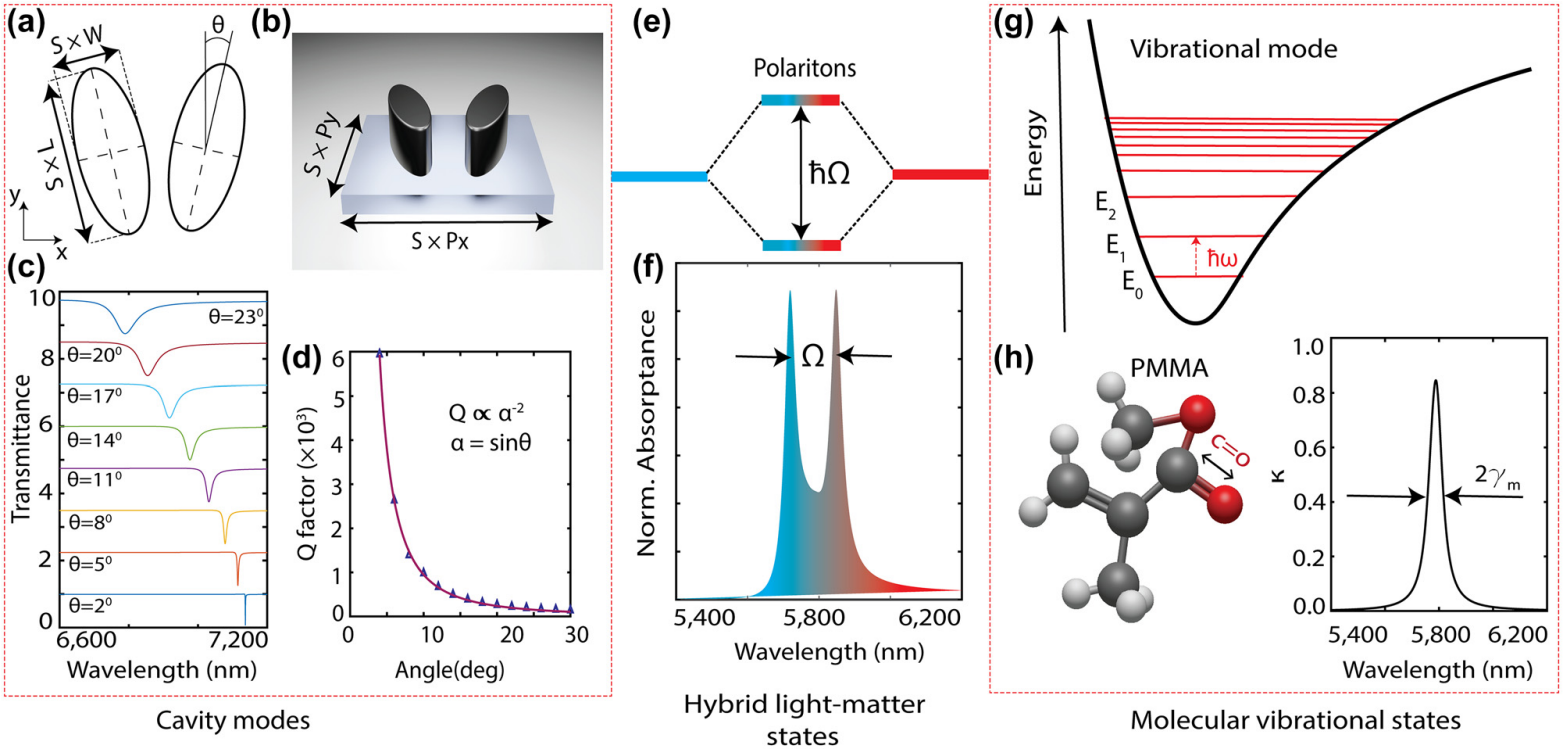
Overview of vibrational strong coupling between a photonic cavity mode and molecular vibrational states. (a and b) Top-view schematic and a rendered image of the metaunit, consisting of two elliptical dielectric structures made of germanium (Ge). The metaunit lies in the *x*–*y* plane with period *Px* = 4050 nm, *Py* = 2340 nm, *L* = 1077 nm, and *W* = 500 nm, representing the long and short axes of the elliptical disks. Scaling parameter *S* and asymmetry parameter *θ* tune the resonance wavelength and *Q*-factor, respectively. (c) Numerically simulated metasurface transmission spectra as a function of the asymmetry parameter *θ*. (d) *Q*-factor of the q-BIC modes as a function of the asymmetry parameter *θ*, revealing adjustability of the photon lifetimes. The solid red line and blue triangles represent the fitted and calculated *Q* factors, respectively. (e) Vibro-polaritonic states distinctly separated by the splitting energy (ℏΩ). (f) Absorption spectra illustrating the formation of splitting under strong coupling. (g) A schematic energy level diagram depicting the molecular vibrational states. (h) The chemical structure of polymethyl methacrylate (PMMA) molecule and its extinction coefficient, corresponding to C=O stretching of its carboxyl group.

In this work, we explore the dynamics of VSC between q-BIC resonances in the MIR over a large parameter space, including cavity *Q*-factor (linewidth), intrinsic molecular losses and transition dipole moment strengths, material thickness filling the metasurface cavity, and the coupling efficiency between cavity mode and the molecular transitions. In our numerical investigations, we focus on the light–matter interactions between germanium (Ge) q-BIC metasurface with the well-known zigzag design [7] and the absorption band associated with the C=O stretching of the PMMA molecule (Figure 1). Our findings reveal the parameter-dependent transition from weak to strong coupling regimes in a coupled system. We specifically leverage the giant yet tunable, resonance lifetimes of the q-BIC modes to demonstrate their substantial effects on the coupled cavity-molecule system. We show that the maximum polariton lifetime can be achieved across various material systems by tuning the cavity photon lifetime and spectral position to the molecule’s unique two-level vibrational resonance. Furthermore, our analysis delves into the practical aspects of spectral polariton detection within a coupled system, revealing that a smaller cavity linewidth facilitates easier separation of polariton peaks. Overall, our results reveal the promising capabilities of the powerful photonic nanocavities supported by the q-BIC dielectric metasurface for studying light–matter interactions while providing design guidance for VSC applications addressing the growing interest in the emerging polaritonic devices field.

## 2 Results

### 2.1 Spectral characterization of VSC

Figure 1a and b depict schematics of the studied metasurface structure, consisting of a zigzag array of Ge elliptical rods on a CaF_2_ substrate. In our investigations, we used a Ge metasurface as it has previously been used for VSC [37]; however, our results also apply to other dielectric metasurfaces, such as Si due to its low loss in the MIR, as demonstrated recently [40]. In this metasurface design, an asymmetry is introduced by tilting the two rods in a metaunit oppositely by *θ* degrees from their long vertical axes, which align with *y*-axis at *θ* = 0 degrees. Thus, the induced in-plane asymmetry disrupts the ideal BIC condition and opens coupling channels to excite the q-BIC modes with horizontally polarized and normally incident plane waves. Since Ge is a low-loss material in the MIR, the radiation losses, which are controlled by the tilting angle, *θ*, determine the cavity linewidth. Figure 1c and d shows the typical inverse quadratic dependence of the cavity *Q*-factor of q-BIC modes to the asymmetry parameter, *θ*.

Moreover, spectral tunability is achieved via a dimension scaling factor, *S*. In this work, we performed electromagnetic simulations using a finite-element frequency-domain solver (CST Studio Suite 2023) with Floquet periodic boundary conditions to study the light–matter interactions on the q-BIC metasurface. The optical constants for the Ge and CaF_2_ were taken from Ref. [41], [42].

The coherent dynamics of a molecule-cavity system can be modeled as two harmonic oscillators interacting with a coupling strength of *g*. When the complex frequencies of molecule (*ω*_*m*_ − *iγ*_*m*_) and cavity (*ω*_*c*_ − *iγ*_*c*_) are considered to account for the damping processes, the eigenfrequencies of the coupled system can be stated as in equation (1).

(1) $$\omega_{\pm }=\frac{\omega_{c}+\omega_{m}}{2}-i\frac{\gamma_{c}+\gamma_{m}}{2}\pm \sqrt{g^{2}+{\left(\delta +i\frac{\left(\gamma_{c}-\gamma_{m}\right)}{2}\right)}^{2}}$$

where *δ* = *ω*_*c*_ − *ω*_*m*_ is the detuning between cavity resonance and molecular vibration modes, and *γ*_*c*_ and *γ*_*m*_ are the cavity and material linewidths, respectively [43], [44]. At nonzero detuning (*δ* ≠ 0), the splitting, defined as the difference between the eigenfrequencies, can be expressed as in equation (2).

(2) $$\Omega =\omega_{+}-\omega_{-}=2\sqrt{g^{2}+{\left(\delta +i\frac{\left(\gamma_{c}-\gamma_{m}\right)}{2}\right)}^{2}}.$$

(3) $$\Omega_{0}=\sqrt{4g^{2}-{\left(\gamma_{c}-\gamma_{m}\right)}^{2}}.$$

At the zero detuning resonance case (*δ* = 0), the difference between the eigenfrequencies becomes equation (3) and known as Rabi splitting, which takes real values when $2g>\left|\gamma_{c}-\gamma_{m}\right|$ . However, this theoretical condition does not guarantee observation of the two distinct polariton modes in practice. To observe strong coupling in an experimental system, the splitting frequency (Ω) needs to exceed the polariton bandwidth, which is the average of the cavity and molecular decay rates (i.e., $\Omega >\frac{\gamma_{c}+\gamma_{m}}{2}$ ). Together, these two conditions impose the strong coupling criterion as $g>\sqrt{\left(\gamma_{c}^{2}+\gamma_{m}^{2}\right)/2}$ .

**Figure 2:**
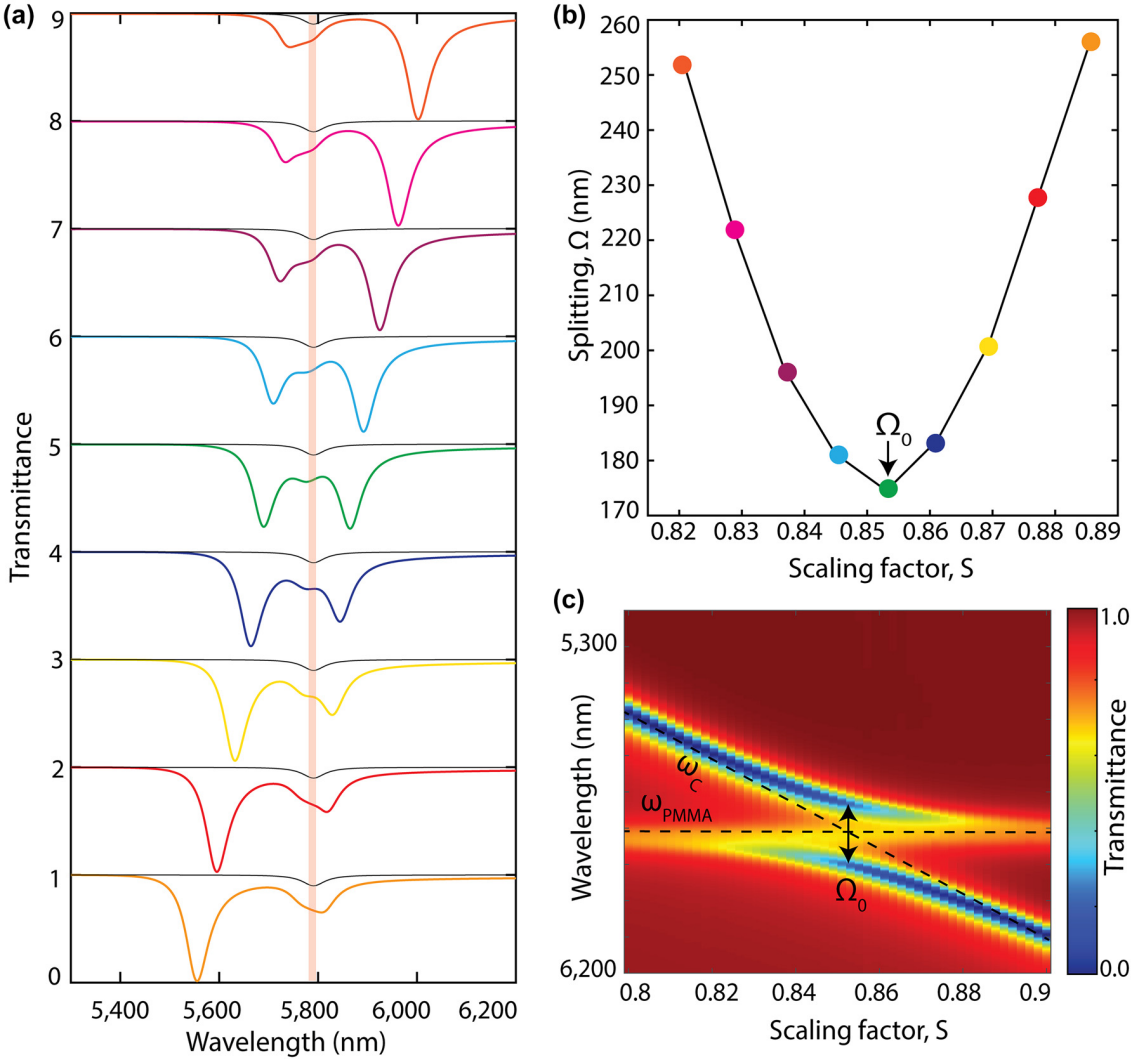
Spectral detuning between cavity resonance and molecular vibration transition mode. (a) Numerically simulated transmission spectra of a coupled system at various detuning where q-BIC metasurface cavity (*θ* = 20°) resonance is spectrally swept through 50 nm PMMA’s C=O absorption peak at 5780 nm resonance wavelength by varying the metasurface scaling factor, *S*. The transmission spectra of 50 nm PMMA layer without the metasurface is shown with the gray line. (b) Splitting, Ω, between the hybrid modes (*ω*_±_) as a function of metasurface scaling parameter, *S*, where Ω_0_ marks the splitting at the zero-detuning (Rabi splitting). (c) Transmission spectra of the coupled system, numerically calculated by varying the metasurface scaling factor, *S*, reveal the hybrid mode anticrossing feature of the VSC. The uncoupled molecule’s vibrational frequency, *ω*_*PMMA*_, and cavity resonance mode, *ω*_*c*_, are individually marked with black dashed lines.

In this work, to study the resonant light–matter interactions, we spectrally sweep the q-BIC resonance by changing the *S* parameter through the molecular vibration mode of the PMMA molecule (Figure 2). Figure 2a demonstrates the impact of spectral detuning (*δ*) within the coupled system. When *δ* is nonzero, the hybrid mode frequencies, *ω*_±_, exhibit asymmetry across the molecular vibration mode, and the anticrossing feature appears (Figure 2c), which is a requisite for the strong coupling. At zero detuning (*δ* = 0), the coupled system is characterized by the Rabi splitting $\Omega_{0}=\sqrt{4g^{2}-{\left(\gamma_{c}-\gamma_{m}\right)}^{2}}$ , which designates the quantitative coupling strength (Figure 2b). In the following analysis, the Rabi splitting in each cavity-coupled material system is identified by polynomial fitting of the simulated hybrid mode splitting values as a function of *S*, and then assigning the minimum of this polynomial as the value at zero detuning Ω_0_ (Figure 2b).

### 2.2 Cavity linewidth and material thickness

The coupling constant between a single two-level vibrational transition system and a cavity is defined by *g*_0_ = *μ*·E_*c*_, where *μ* is molecule’s transition dipole moment and *E*_*c*_ is the cavity vacuum electric field. According to this relation, the cavities that can better localize and enhance the density of states in a cavity can more efficiently couple to molecular transitions. Moreover, when more than *N* molecules couple to the same cavity resonance, the collective coherence between the molecular ensemble and the cavity increases by a factor of $\sqrt{N}$ to $g=\sqrt{N}g_{0}$ (from Ref. [45]).

**Figure 3:**
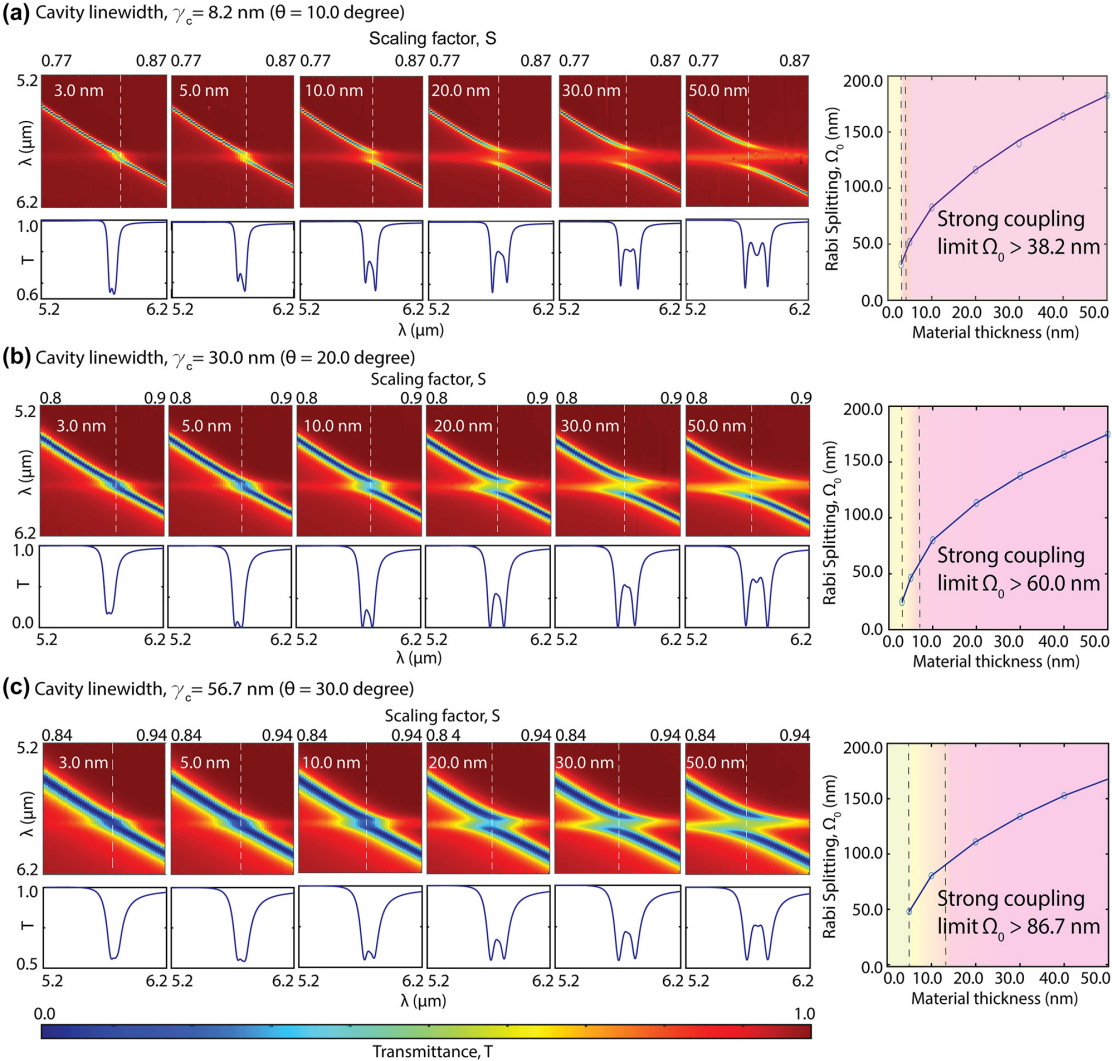
Effects of cavity linewidth and material thickness on the coupled system. The material thickness effect is shown for three different cavity linewidths with (a) *γ*_*c*_ = 8.2 nm achieved by *θ* = 10°, (b) *γ*_*c*_ = 30 nm achieved by *θ* = 20°, and (c) *γ*_*c*_ = 56.7 nm achieved by *θ* = 30°. For each cavity linewidth, the transmission spectral maps as a function of scaling factor (i.e., various detuning), *S*, are presented for different PMMA layer thicknesses (3.0 nm, 5.0 nm, 10.0 nm, 20.0 nm, 30.0 nm, and 50.0 nm), showing the anticrossing patterns. Under each spectral map, a single transmission spectrum, *T*, at zero detuning point is shown. The white dashed lines indicate the zero detuning points for each coupled system. On the right column, plots show the Rabi splitting at the zero detuning, Ω_0_, as a function of material thickness. From (a) to (c), as the metasurface cavity linewidth increases, the weak-to-strong coupling transition shifts to higher material thicknesses, indicating the requirement of a larger number of collectively coupled molecules (*N*) for VSC to compensate for the cavity’s photon loss. The newly defined transition region is demonstrated with yellow and yellow-to-pink transition colors, and the strong coupling regime is indicated by pink color.

To evaluate the ensemble molecule (*N*) effect on the coupling strength, we calculated the Rabi splitting as a function of PMMA material thickness on a metasurface. By varying the PMMA layer thickness (*t* = 3, 5, 10, 20, 30, and 50 nm), we controlled the total number of molecules (*N*) within a single cavity. Moreover, we repeated this study for three different q-BIC modes with varying cavity linewidths, *γ*_*c*_ = 8.2, 30, and 56.7 nm, achieved by increasing the tilting angle, *θ* = 10, 20, and 30°, respectively (Figure 3). On the right column of Figure 3a–c, we plot the Rabi splitting values calculated for each material thickness. The findings depicted in Figure 3 demonstrate the $\sqrt{N}$ relation between the Rabi splitting and the PMMA layer thickness, as expected.

Notably, the coupling strength signified by the Rabi splitting versus the material thickness relation is not identical in cavities with different photon damping characteristics (Figure 3a–c). We calculated the strong coupling criteria for each cavity linewidth, *γ*_*c*_ = 8.2, 30, and 56.7 nm, with Ω_0_ > 38.2 nm, 60.0 nm, and 86.7 nm, respectively. Our investigation in Figure 3 revealed that the minimum material thickness required to satisfy the strong coupling condition increases with the cavity linewidth, associated with the total cavity photon losses. The simulation results in Figure 3a shows that the strong coupling condition can be satisfied with as thin as 3 nm PMMA when the q-BIC mode is sharp with 8.2 nm linewidth. However, an increase in material thickness is necessary to achieve strong coupling when the cavity linewidth increases from 8.2 nm to 30.0 nm and 60.0 nm. With the higher loss cavities (larger *γ*_*c*_), when the total molecule number (*N*) is low, the ensemble coupling strength (*g*) is not sufficient for strong coupling, and the light–matter interactions are characterized under the weak coupling condition ( $g\leq \sqrt{\left(\gamma_{c}^{2}+\gamma_{m}^{2}\right)/2}$ ). The weak coupling condition is usually characterized by a resonance peak damping and quantified by the photonic resonance amplitude decrease in the presence of absorbing molecules [11].

However, the transition between weak and strong coupling is usually not abrupt. Therefore, here, we define a new “transition coupling region,” where the polaritons can be observed in the spectrum as the Rabi frequency has a real but small value ( $\left|\Omega_{0}\right|>0$ ), even though the strong coupling criterion is not met. For example, in Figure 3a, the presence of hybridized mode splitting in the transmission spectra (lower subfigure) is observable even for 3 nm material thickness. This cavity material system does not satisfy the strong coupling condition; however, the spectrum diverges from a simple mode damping scheme, which is a characteristic of weak coupling. Thus, we define the new transition region condition as $\left|\Omega_{0}\right|>0\;\text{and}\;g\leq \sqrt{\left(\gamma_{c}^{2}+\gamma_{m}^{2}\right)/2}$ ).

### 2.3 Material parameters

To reveal the dependency of the VSC on the molecular vibrational resonance properties, we studied various artificial materials, which are derived from the C=O bond stretching resonance in the PMMA molecule (PMMA linewidth ∼50 nm). The complex permittivity of these materials is calculated using the Lorentz model for dispersion in dielectrics [46]:

$$\varepsilon \left(f\right)=\varepsilon_{\infty }+\frac{\Delta \varepsilon f_{0}^{2}}{f_{0}^{2}-2jf\gamma_{m}-f^{2}}$$

where *ɛ*_*∞*_ is the background relative permittivity, *f*_0_ is the Lorentz resonance frequency, Δ*ɛ* is the absorbance strength coefficient, and *γ*_*m*_ the Lorentz damping rate of the molecule. Some realistic parameter values used to model the artificial materials are provided in Tables 1 and 2. Coupling the designated materials to the q-BIC cavities allowed us to study correlations between Rabi energy exchange rate and materials’ absorption strength and nonradiative losses. In IR absorption spectroscopy, the amplitude of absorption depends on the polarity of the bond, as well as the concentration of the bonds in a molecule. Therefore, the same functional group can have different absorption strengths (Δ*ɛ*) depending on the molecule. Moreover, when collecting IR spectra from a compound in liquid or solid phase, many individual molecules interact with each other. Thus, individual bond absorptions occur at varying frequencies, broadening the absorption peaks at different levels. The dephasing due to electron–phonon interactions can be reduced by cooling the sample to cryogenic temperatures, which can reduce material loss (*γ*_*m*_). In our strong coupling investigations, we consider these realistic material properties to interrogate each designated material’s cavity-coupled interactions.

**Table 1:** Lorentz oscillator parameters used to design three artificial materials with different absorption strength Δ*ɛ* with the same damping rates *γ*_*m*_. The imaginary parts of the permittivity for the three designated materials are plotted around the resonance frequency (*f*_0_), showing the characteristic Lorentzian spectral line shape with fixed linewidth.

**#**	***ɛ*_*∞*_**	***γ*_*m*_** **(THz)**	***f*** **_0_** **(THz)**	**Δ*ɛ***	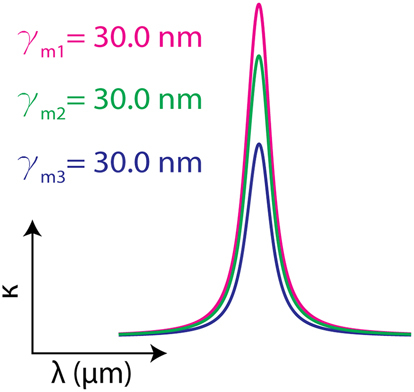
1	2.198	0.235	51.77	0.025	
2	2.198	0.235	51.77	0.050	
3	2.198	0.235	51.77	0.070	

**Table 2:** Lorentz oscillator parameters used to design three artificial materials with different damping rates *γ*_*m*_ while keeping their absorption strengths Δ*ɛ* the same. The imaginary parts of the permittivity for the three designated materials are plotted around the resonance frequency (*f*_0_), showing the characteristic Lorentzian spectral line shape with varying linewidth.

**#**	***ɛ*_*∞*_**	***γ*_*m*_** **(THz)**	***f*** **_0_** **(THz)**	**Δ*ɛ***	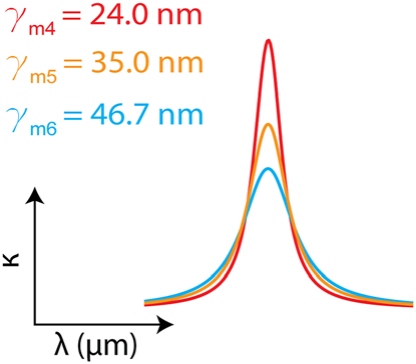
4	2.198	0.10	51.77	0.009	
5	2.198	0.15	51.77	0.009	
6	2.198	0.20	51.77	0.009	

**Figure 4:**
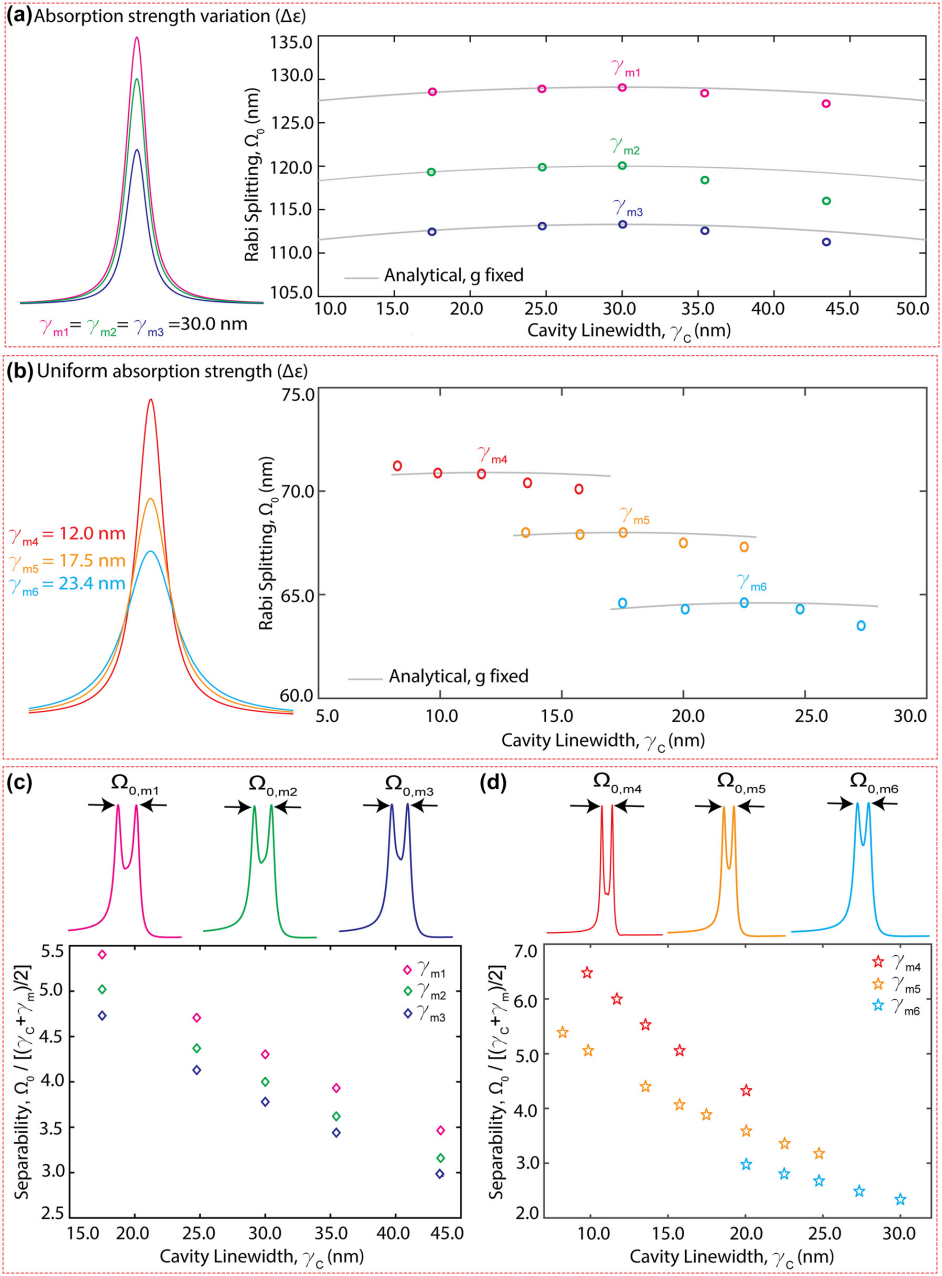
Effects of material loss and absorption strength on the coupled system. (a) Numerically calculated Ω_0_ as a function of cavity linewidth, *γ*_*c*_, for three different materials with the same absorption linewidth but different absorption strength. (b) Numerically calculated Ω_0_ as a function of cavity linewidth, *γ*_*c*_ for three different materials with different damping rates but same absorption strength. The solid gray lines in (a) and (b) denote the analytical Ω_0_ calculated with a fixed coupling strength, *g* (equation (3)). Analysis of polariton peak separability as a function of cavity linewidth, *γ*_*c*_, observed across materials used in (a) and (b) is shown in (c) and (d), respectively. The polariton spectra with Rabi splitting Ω_0,*m*1_ to Ω_0,*m*6_ corresponding to the materials *γ*_*m*1_ to *γ*_*m*6_ at critical coupling are also shown.

In Figure 4, we present our findings on the coupling strength as a function of material properties and the cavity linewidth. Figure 4a shows the Rabi splitting for three materials with different absorption strengths (Δ*ɛ*) and the same damping rates (*γ*_*m*_) as a function of the cavity linewidths (*γ*_*c*_ = 10–50 nm) that they couple to. Since the absorbance peak is associated with the molecule’s transition dipole moment (*μ*), which determines the coupling constant ( $g=\sqrt{N}\left(\mu \cdot E_{c}\right)$ ), the total Rabi splitting at zero detuning Ω_0_ (equation (3)) increases with the material absorbance strength. Moreover, when the damping rates of the material and the cavity are equal (*γ*_*c*_ = *γ*_*m*_), the critical coupling condition is met, maximizing the strong coupling efficiency at Ω_0_ = 2*g*. In Figure 4b, the Rabi splitting is depicted for three materials characterized by varying damping rates (*γ*_*m*_) but uniform absorption strengths (∆ε), as a function of the cavity linewidths (*γ*_*c*_ = 5–30 nm) to which they are coupled. The material with the smallest damping rate (*γ*_*m*4_) exhibits the highest absorption peak among the three materials, because of the lower loss.

Further, we analyzed the cavity linewidth effects on the separability of polariton peaks in a strongly coupled system considering the materials defined in Tables 1 and 2. Here we defined polariton separability as $\frac{\Omega_{0}}{\left(\gamma_{c}+\gamma_{m}\right)/2}$ , which measures how easily polaritons can be identified in an experimental spectral analysis. As expected, narrower material linewidths facilitate better separability in any material system (Figure 4c and d). The representative polariton spectra at critical coupling presented on top of these figures illustrate our findings. Moreover, our investigation revealed that larger absorption strength facilitates the polariton peak separation due to higher Ω_0_ (Figure 4c). Furthermore, materials with the smallest nonradiative losses (small *γ*_*m*_) maximize the polariton separability (Figure 4d). These observations elucidate the pivotal role played by material characteristics, particularly the linewidth and absorption strength, on the separability of polariton peaks within a coupled system. The interplay between the material and the cavity properties sheds light on the multifaceted attributes of identifying polariton peaks in experimental investigation.

### 2.4 Cavity-material coupling efficiency

**Figure 5:**
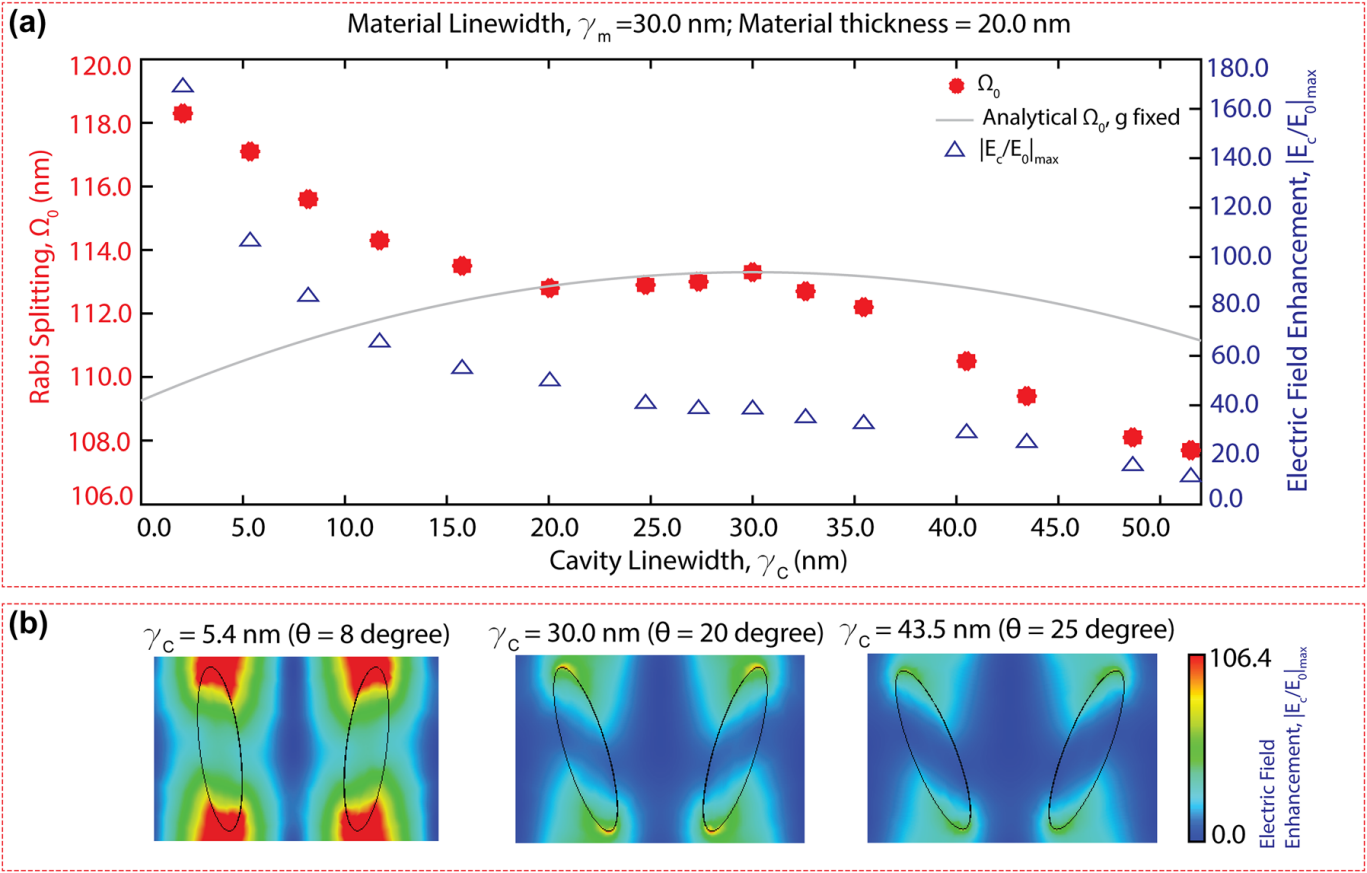
Critical strong coupling and cavity loss effects on the light–matter interactions. (a) For the same material type and thickness, the calculated Ω_0_ (denoted by red asterisk) is plotted as a function of cavity linewidth, *γ*_*c*_. The gray line shows the analytically calculated Rabi splitting as a function of *γ*_*c*_, assuming a constant coupling strength, *g* (equation (3)). The maximum field enhancement indicated by the blue triangles is plotted as a function of *γ*_*c*_. (b) The electric field enhancement maps for three different metasurface cavities are depicted with reference to the same color bar.

Furthermore, we investigated the coupling strength within the hybrid system by adjusting the *Q*-factor of the q-BIC cavity mode. In Figure 5(a), we present Ω_0_ (red asterisks) values calculated by simulating a 20 nm thick PMMA with a linewidth of *γ*_*m*_ = 30 nm on a q-BIC metasurface whose cavity linewidth was swept from *γ*_*c*_ = 2–52 nm by varying the tilting angle, *θ* from 5 to 28°. The gray line shows the analytically calculated equation (3), as a function of the cavity linewidth (*γ*_*c*_), assuming a constant material damping (*γ*_*m*_) and constant coupling strength, *g*. The parabolic dependency of Ω_0_ on the cavity loss displays how the coupling efficiency reaches a local maximum when the critical coupling (*γ*_*c*_ = *γ*_*m*_) condition is satisfied and how the loss mismatch between the cavity and the material decreases the coupling efficiency. The discrepancy between the simulation (red asterisk) versus analytical (gray line) results underscores *γ*_*c*_, affects $g=\sqrt{N}\left(\mu \cdot E_{c}\right)$ , by changing the field enhancement of the bare metasurface (blue triangles). As *γ*_*c*_ decreases, E-field enhancement increases, and thus boosting *g*, which in turn increases Ω_0_. This is further illustrated in Figure 5b, where we present the absolute electric field enhancement maps ( $\left|E_{c}/E_{0}\right|$ ) within a bare metaunit for three q-BIC metasurfaces whose tilting angle is adjusted to yield *γ*_*c*_ of 5.4 nm (*θ* = 8°), 30 nm (*θ* = 20°) and 43.5 nm (*θ* = 25°). As the maps show, the cavity field becomes stronger as *γ*_*c*_ decreases (i.e., metasurface *Q*-factor increases). As expected, the metasurface cavity resonance gets perturbed by the presence of lossy materials, and thus the effective field enhancement decreases when the cavity is coated with materials (Figures S1 and S2). The interplay between the cavity quality factor and photon loss rate mismatch between the cavity and material provides crucial insights into the dynamics governing the coupling behaviors in a hybrid system.

**Figure 6:**
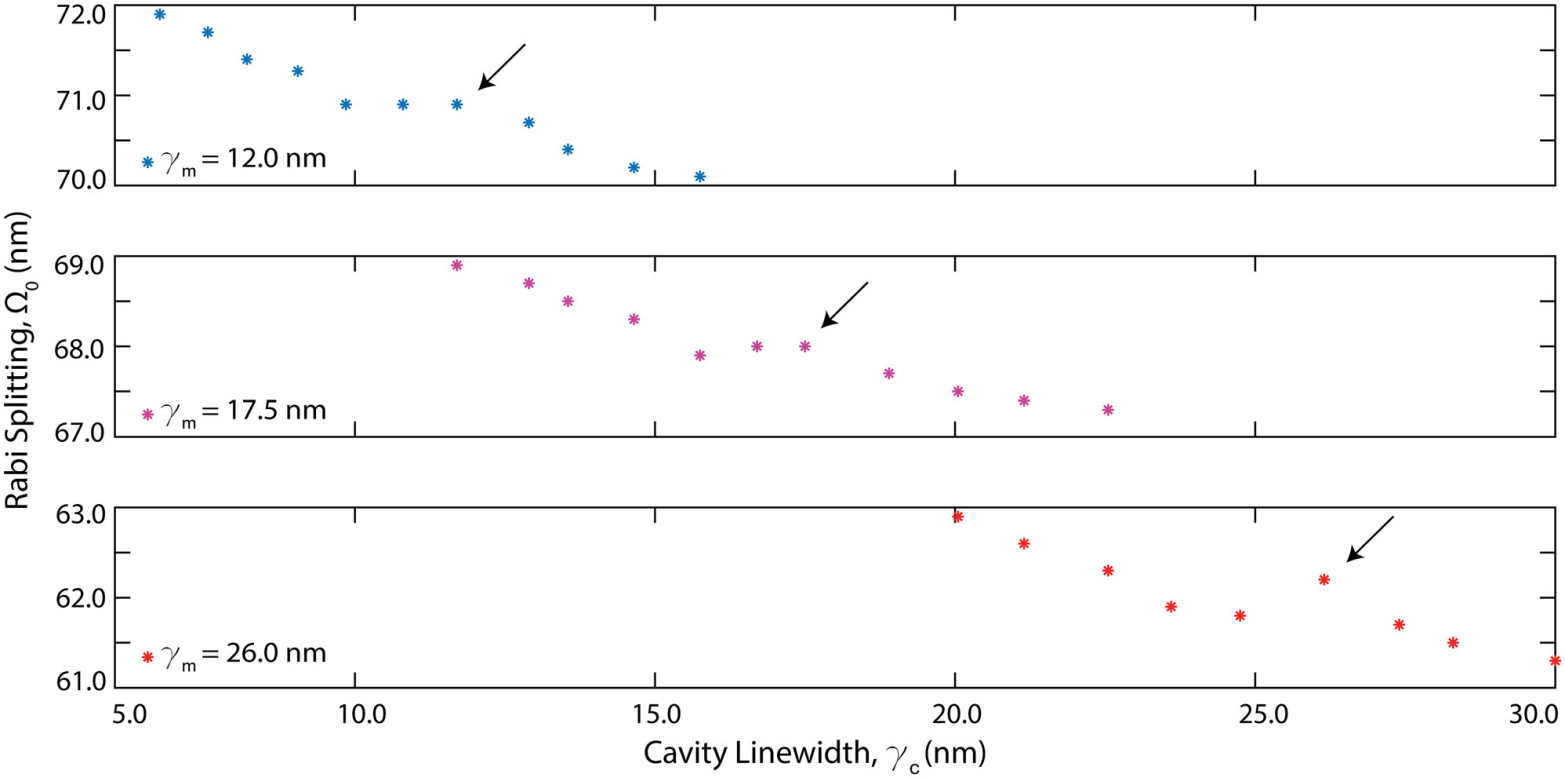
Critical strong coupling and material loss effects on the light–matter interactions. Rabi splitting as a function of cavity linewidth plotted for three different materials. As the material loss decreases (*γ*_*m*_ = 26 nm to *γ*_*m*_ = 12 nm), critical strong coupling shift toward the high-Q cavity region, causing an increase in coupling strength, *g*, and boosting the Ω_0_. Further boost of Ω_0_ is also achieved at critical coupling as shown with the black arrows.

Finally, we explored the critical coupling effects for different material-cavity systems. Figure 6 shows the Rabi splitting (Ω_0_) variation as a function of cavity linewidth (*γ*_*c*_ = 5–30 nm) for three different materials (*γ*_*m*_ = 12.0 nm, 17.5 nm, and 26.0 nm). Our results reveal that it is favorable to work with ultrahigh-Q cavities to achieve strong coupling independent of the material’s vibrational transition characteristics. However, when real-world metasurface fabrication and intrinsic material losses limit the maximum achievable cavity resonance *Q*-factors, satisfying the critical coupling condition by cavity-material loss matching can also enhance the coupling strength (as indicated by the black arrows). This effect is specifically pronounced in materials with large absorption linewidths (see Figure 6 bottom). Overall, Figure 6 unveils how the *Q*-factor tunability of q-BIC cavities supported by resonant dielectric metasurface is important for VSC applications.

## 3 Discussion

This work was initially motivated by the two published prominent reports using similar dielectric metasurface supporting the q-BIC resonance modes based on the zigzag geometry. In the work by Tittl et al., the light–matter interactions in the weak coupling regime were leveraged for biochemical sensing of proteins and polymer layers [11]. Meanwhile, Sun et al. experimentally demonstrated strong vibrational coupling with PMMA layers using similar metasurfaces [37]. Therefore, to elucidate the underlying physical parameters that determine the light–matter interaction regime, in this work, we extensively investigated the resonant cavity parameters of the zigzag metasurface design, such as the *Q*-factor (tuned by the symmetry-breaking factor, *θ*) and spectral position of the resonance (tuned by the scaling factor, *S*). We also considered material parameters, such as the absorption bandwidth related to the molecules’ dephasing and internal losses, transition dipole moment strength, and material thickness filling the cavity void. Our findings unveiled that as the metasurface *Q*-factor increases, the minimum number of molecules in a single cavity (material layer thickness on a metasurface) requirement for VSC decreases. This collective molecule-cavity coupling effect explains the weak coupling regime in Tittl et al., where thin protein monolayers were measured, and the strong coupling regime in Sun et al., where 10 s of nm thick polymer layers were measured [37].

Moreover, previous VSC investigations mainly used photonic cavities where the cavity photon loss rates were dominantly imposed by the cavity materials, specifically metals’ ohmic losses, such as in the FP and plasmonic cavities. Therefore, the effects of the photonic cavity’s *Q*-factor on the light–matter interactions were mostly unexplored in the MIR. Recently, all-dielectric metasurface introduced unprecedented efficiency in photon trapping into nanocavities, diminishing the limitations of cavity loss. Notably, metasurface supporting the q-BIC modes enabled the precise control of cavity photon loss rates into radiation channels via the geometrically tunable in-plane asymmetry parameters. Thus, in this work, we outlined the benefits of cavity *Q*-factor tunability on the light–matter coupling efficiency.

As a rule of thumb, when using dielectric metasurface for VSC applications, the first goal must be to minimize the metasurface material and radiation losses to maximize the cavity *Q*-factor, which increases the resonance field enhancement and thus the coupling constant, *g*. However, in real-world applications, metasurface fabrication imperfections, together with nonzero material loss rates, constrain the practically achievable cavity *Q*-factors. Then, a second important criterion for VSC and efficient light–matter interactions is the material and cavity loss matching. Uniquely, dielectric metasurface can enable critical strong coupling conditions via their *Q*-factor tunability. It is also noteworthy that the arguments we presented regarding *Q*-factor and coupling strength applies only to structures where their *Q*-factor is directly related to their mode volume size. Structures such as Distributed Bragg Reflectors (DBR) can also present high *Q*-factors, yet with relatively large mode volumes. Moreover, in the case of DBR, the field enhancement region is contained within the structure and thus limits mode interaction with target molecules.

Generally, strong coupling is defined based on the Rabi splitting exceeding the system loss rate, enabling coherent energy exchange between the cavity and the molecule. However, the literature has been ambiguous on the quantitative limits of the strong coupling regime. In this work, we adhered to the condition where the exchange rate must exceed the average loss of the two coupled oscillators ( $\Omega_{0}>\left(\frac{\gamma_{c}+\gamma_{m}}{2}\right)$ ). Others have proposed a strong coupling criterion where Rabi energy exchange exceeds the larger of the two loss rates (Ω_0_ > *γ*_*c*_, *γ*_0_). While the polaritons form when $\left|\Omega_{0}\right|>0$ , where Ω_0_ is small and polariton linewidth is large, spectroscopic interrogation of hybrid states depends on the experimental optical measurement limitations. Thus, in this work, we named this ambiguous condition as a “transition region” where hybrid modes are present ( $\left|\Omega_{0}\right|>0$ ), but strong coupling condition is not strictly satisfied ( $\Omega_{0}\leq \left(\frac{\gamma_{c}+\gamma_{m}}{2}\right)$ ). For example, our findings revealed that materials with narrower linewidths and higher absorption peaks coupled to high-Q cavities enable clearer spectral peak separability. Usually, by leveraging the collective coupling (high *N*), Rabi splitting can be increased to bring the system to the strong coupling regime for spectral investigations. Exceptionally, our findings lay the compelling research question of whether transition region can be the zone where the polaritons from single or low counts of molecules’ strong coupling to a cavity can be measured.

## 4 Conclusions

In this work, we used CST simulation tools and coupled oscillator theory to numerically and analytically explore the cavity-coupled light–matter interactions on q-BIC metasurface. We comprehensively outlined the parameter space vital for the understanding of the coupling between resonant cavity modes and resonant vibrational transitions in molecules. We leveraged the geometrical *Q*-factor and spectral position tunability of the q-BIC resonances to identify the weak and strong coupling regimes, providing a deeper insight into the interaction dynamics within the coupled system. Our findings indicate that, to satisfy the VSC condition in lossy cavities, collective mode coupling to a metasurface resonance is necessary, and minimum number of required molecules for VSC increases as the cavity *Q*-factor decreases. Notably, we introduced a new transition light–matter coupling zone, which falls in between the weak and strong coupling extents, where polaritons form, but their linewidth prohibits their spectral identification. Moreover, we studied molecules’ transition dipole strength, absorption bandwidth correlated to their internal loss, and the total number of molecules coupled to a single cavity, to gauge their individual effects on the light–matter interactions. Our results can provide clear guidelines for designing optimal dielectric metasurface for VSC applications using a broad range of molecules’ vibrational transitions. Based on our comprehensive study, we anticipate that the photonic cavities generated by dielectric metasurface can make a huge impact in the emerging field of polaritonic chemistry, where ground states and chemical reactivity of molecules can be modified.

## Supplementary Material

Supplementary Material Details
