# Supplementary material for: From weak to strong coupling: quasi-BIC metasurfaces for mid-infrared light–matter interactions
Source: Nanophotonics. 2024 Apr 15;13(16):2937–49. doi: 10.1515/nanoph-2024-0043 (PMC11245121; doi:10.1515/nanoph-2024-0043)
Supplement: Supplementary file 1 — Supplementary Material Details [file j_nanoph-2024-0043_suppl_001.docx]

Supporting Information

**From Weak to Strong Coupling: Quasi-BIC Metasurfaces for Mid-infrared Light-Matter Interactions**

Shovasis Kumar Biswas^a^†, Wihan Adi^b^†, Aidana Beisenova^b^, Samir Rosas^b^, Eduardo Romero Arvelo^a^, Filiz Yesilkoy^b^*

^a^ Department of Electrical and Computer Engineering, University of Wisconsin-Madison Madison, WI 53706, USA

^b^ Department of Biomedical Engineering, University of Wisconsin-Madison Madison, WI 53706, USA

*Email: filiz.yesilkoy@wisc.edu

† Authors contributed equally

The field enhancement values shown in fig. 5a are calculated from the bare metasurface and serves as a relevant metric associated with the original field localization capabilities of the metasurfaces. The coupling of the materials with the cavity can be considered as a perturbation to the cavity resonance, and thus decreases the field-enhancement. Especially, by tuning the cavity resonance to the absorption peak of a molecule, we are coupling a photonic cavity to a lossy material. To show how field-enhancement changes with weak and strong coupling, we further investigated the cavity with $\theta$ = 20 degree from Fig. 3b, where we identified a weak coupling with 3 nm of material and a strong coupling with 50 nm of the same material in the same cavity. In the following two figures (S1 and S2), we show that the field-enhancement of the uncoated metasurface has the highest overall values throughout space, and this field enhancement decreases as the coupling strength to a lossy material increases via increasing the material quantity in the cavity.


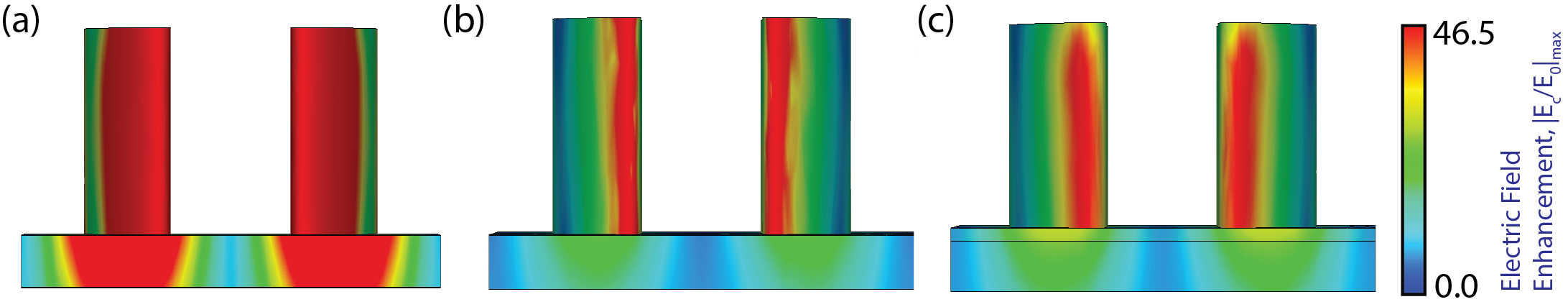


**Fig. S1:** Electric field enhancement maps showing three metasurface cavities with a fixed tilting angle of θ = 20 degrees and different material thicknesses: (a) no material, (b) 3 nm thick material (weak coupling), and (c) 50 nm thick material (strong coupling).


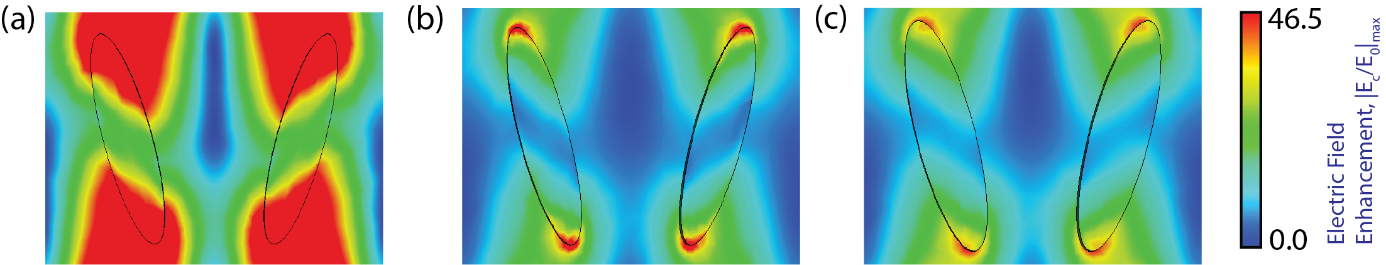
**Fig. S2:** Electric field enhancement maps showing three metasurface cavities with a fixed tilting angle of θ = 20 degrees and different material thicknesses: (a) no material, (b) 3 nm thick material (weak coupling), and (c) 50 nm thick material (strong coupling).
